# Empowering chronic disease management with smart healthcare in China: a policy effectiveness evaluation by PMC index model

**DOI:** 10.3389/fpubh.2026.1729450

**Published:** 2026-02-17

**Authors:** Hang Yang, Xi Wang, Tao Zhang, Shufang Zhao, Rongjiang Cai, Dongyang Wang

**Affiliations:** 1 Faculty of Humanities and Social Sciences, Macao Polytechnic University, Macau, China; 2 Department of Nursing, The Third People's Hospital of Henan Province, Zhengzhou, China

**Keywords:** chronic disease, health policy assessment, PMC index model, smart healthcare, text mining

## Abstract

**Introduction:**

Population aging has driven a sustained increase in chronic diseases, creating major challenges for management, while the development of smart healthcare technologies offers new approaches for chronic disease prevention and control. Systematic analysis of existing policies on intelligent chronic disease management remains insufficient in China, necessitating detailed scrutiny of such policies.

**Methods:**

This study systematically reviewed China's national-level policies on chronic disease management over the past decade, using the PMC index model and text mining techniques to analyze the policy texts and construct a comprehensive evaluation index system for intelligent chronic disease management.

**Results:**

The 18 policies analyzed focus on chronic disease management, data sharing, telemedicine and multi-party collaboration. The average PMC index score of the policies is 7.31 (on a 0–10 scale). Among them, five policies are rated as “excellent” and 13 as “good,” reflecting a generally high overall quality, with P14 performing the best. However, the implementation details and regulatory framework of the policies are still vague, and there are large differences in the implementation progress between regions.

**Conclusion:**

These existing policies align well with the requirements of contemporary medical development and are conducive to improving the quality of chronic disease management and its effectiveness, but ambiguities and deviations still exist in the policy implementation, requiring more specific classification and guidance. Future work should target these gaps to improve patient care outcomes.

## 1 Introduction

Chronic diseases have become a global public health challenge, posing a serious threat to human health and life quality (1, 2). According to the Global Burden of Disease (GBD) study, chronic diseases are the main cause of death worldwide, accounting for over 60% of all deaths (3). Many countries are exploring chronic disease management models in order to address this challenge. In the United States, where chronic diseases are the leading cause of death and disability and thus place a heavy burden on healthcare resources. In response, multiple approaches have emerged, including chronic care models and peer support models (4, 5). By integrating healthcare resources, strengthening patient education, and promoting self-management, these models have improved management outcomes to some extent. However, issues such as inadequate health insurance coverage and high medical costs persist. Australia has implemented innovative government subsidy programs, particularly for primary care management of cardiovascular diseases. Leveraging the National Chronic Disease Management Program funded by universal health insurance, general practitioners develop personalized management plans for patients and provide subsidized clinical services. This has effectively increased community control rates for cardiovascular risk factors such as hypertension and diabetes (6). Europe faces the severe challenge of rising chronic disease prevalence and increasing prevention expenditures (7). Through transnational chronic disease economics projects, it has established cost-effectiveness evaluation models, prioritizing the prevention and control of major chronic diseases, such as coronary heart disease and diabetes, along with their associated risk factors. It explores cost-effectiveness methodologies and assesses intervention outcomes while actively advancing the development of the healthcare system and strengthening primary care services.

Beyond Europe and the United States, Asian nations are also taking proactive steps. Japan emphasizes patient self-management and preventive healthcare, enhancing public health literacy and chronic disease awareness through community health promotion activities and health education programs (8). In China, rapid socioeconomic development and accelerated population aging have led to significant shifts in residents' income levels, dietary habits, and lifestyles. The epidemiological pattern has transitioned from predominantly infectious diseases to one dominated by chronic noncommunicable diseases. Chronic diseases are characterized by insidious onset, prolonged course, and complex etiology. Their incidence has continued to rise in recent years, posing severe challenges to chronic disease management systems. The latest research data indicates that the prevalence of chronic diseases among the population aged 60 and above in China has reached 81.1% (9). Chronic diseases account for 80% of all deaths nationwide and over 70% of the disease burden. Among these, major chronic diseases such as cardiovascular and cerebrovascular diseases, cancer, and chronic respiratory diseases severely impact the health status of the population (10). Although China has established a relatively comprehensive healthcare service system and disease prevention and control network, with continuous improvements in residents' health indicators, the field of chronic disease management still faces prominent challenges, such as insufficient service capacity at primary healthcare institutions and weak patient self-management awareness (11). The above domestic and international practices have shown that although traditional management models have achieved certain results, they still face common bottlenecks in dealing with the continuously increasing incidence of diseases, achieving accessibility and precision of services, and controlling long-term medical costs.

In this context, with the rapid development of cutting-edge technologies such as big data, artificial intelligence, and the Internet of Things (IoT), smart healthcare is increasingly becoming the core driving force for global healthcare transformation, providing unprecedented opportunities to optimize medical service processes, improve medical efficiency, and enhance patient health outcomes (12, 13). Smart healthcare, empowered by technology, has the potential to overcome the limitations of traditional models and become a key breakthrough in improving the quality and efficiency of chronic disease management. China places high importance on chronic disease management, incorporating it into the core areas of national strategy. Policy documents such as the “Healthy China 2030” Planning Outline and the “National Medium- and Long-Term Plan for Chronic Disease Prevention and Control (2017–2025)” provide robust policy support for chronic disease management. Governments at all levels have actively responded, forming a coordinated, multi-level governance framework. By promoting family doctor contract services and incorporating chronic disease management into basic public health service programs, China has achieved comprehensive, lifecycle-based health management for all chronic disease patients (14). The establishment of a tiered diagnosis and treatment system has further optimized the allocation of medical resources, enhancing the convenience and quality of care for chronic disease patients (15). Concurrently, China has witnessed continuous innovation in the field of chronic disease prevention and control. Novel drug development, the application of smart medical devices, and innovations such as Internet + healthcare technologies (16, 17) are injecting new momentum into chronic disease management, driving healthcare services toward greater efficiency and precision. However, despite the rich exploration of policies and practices, there is currently a lack of systematic quantitative evaluation of China's smart chronic disease management policy system, resulting in a blank understanding of policy focus, effectiveness, and overall evolution patterns.

Therefore, to fill this research gap, this research conducts a systematic quantitative analysis and evaluation of national-level chronic disease management policy documents issued in China over the past decade. By combining text mining and PMC index model analysis to construct a policy evaluation indicator system, the findings reveal the focal points of China's smart chronic disease management policies and expose existing shortcomings. This provides valuable reference for optimizing China's smart chronic disease management policy framework and advancing research in related fields.

## 2 Literature review

With continuous advances in modern medical technology and rising living standards, chronic diseases have become one of the major public health challenges worldwide. As the world's most populous country, China faces a growing burden of chronic diseases (18), particularly the rising prevalence of cardiovascular diseases, diabetes, and cancer. Consequently, this places immense pressure on the national healthcare system and has progressively become a key factor affecting public health. Chronic disease prevention and control have thus become integral components of national health policy (19).

Since the early 21st century, the Chinese government has placed a high priority on chronic disease prevention and control, implementing a series of policy measures. As early as 2006, the National Health Commission first put forward the concept of chronic disease prevention and control, subsequently advancing the implementation of related policies over the following years. One notable example is the “ China Chronic Disease Prevention and Control Medium- and Long-Term Plan (2017–2025),” which explicitly aims to reduce the incidence and progression of chronic diseases by strengthening early screening, health management, and the application of innovative technologies. It clearly outlines objectives and tasks for chronic disease prevention and control, providing directional guidance for implementing various chronic disease management policies.

In recent years, with the continuous advancement of smart technologies, national support for intelligent health management systems has seen a steady increase (20). Particularly in the field of chronic disease management, policies have increasingly encouraged the application of technologies such as big data and artificial intelligence to enhance management efficiency and effectiveness. The 2016 Guiding Opinions on the “Internet + Initiative” clearly advocated for advancing digital transformation in healthcare, developing online health consultations, and intelligent management services. This laid the policy foundation for the widespread adoption of smart chronic disease management. Furthermore, the Healthy China Initiative (2019–2030), released in 2020, highlighted the paramount significance of health management and technological innovation in chronic disease management. This initiative aims to elevate national health standards through digital means.

Against the backdrop of smart healthcare, China's chronic disease management policies have transitioned from initial exploration to in-depth implementation. Early policies mainly centered on traditional approaches such as health education (21), community interventions (22), and patient self-management (23). As a result of these technological shifts, with technological advancements-particularly the widespread adoption of mobile internet and Internet of Things technologies-intelligent chronic disease management has increasingly emerged as the mainstream (24).

Dhamanti et al. (25) pointed out that smart healthcare not only effectively improves treatment outcomes for chronic disease patients but also reduces healthcare costs by enabling personalized health management through data analysis and intelligent monitoring. The Chinese government encourages the development of smart health devices (such as smart wristbands and smart blood pressure monitors) and telemedicine to achieve real-time monitoring and intervention for chronic disease patients (26, 27). Wearable devices like smart bracelets have made remarkable progress in chronic disease monitoring. Research by Ma (28) indicates that their developed deep learning model achieved an average accuracy rate of 90.1% in health data analysis, significantly surpassing traditional methods, with a 5%−8% improvement in accuracy. These machine learning-based smart bracelets provide more precise daily activity monitoring tools for patients with chronic conditions like hypertension and diabetes, enabling real-time awareness of their health status. Tański et al. (29) investigated the role of telemedicine in monitoring and intervening patients with chronic disease and highlighted that remote connections between data analysis platforms and physicians enabled real-time access to patient health data and timely interventions to prevent or reduce negative consequences.

In recent years, the rapid development of smart health management systems has offered chronic disease patients more sophisticated health management solutions (30). These systems, leveraging digital technologies like electronic health records, mobile health apps, and big data, enable continuous monitoring and personalized interventions for chronic disease patients, which significantly enhances management outcomes (31–33). However, the rapid proliferation of mobile health technologies has also brought challenges. Despite offering substantial convenience, smart health management systems still pose risks in privacy protection and data security. Researchers, including Ni et al. (34), conducted a systematic assessment of personal information protection compliance in 45 mainstream chronic disease management apps in China. They did this by developing scales and establishing multi-level indicators. They found that most apps demonstrate notable shortcomings in privacy protection, highlighting the urgent need for strengthened regulation and technological advancements. Consequently, policy safeguards become particularly crucial in this context.

Quantitative policy text analysis utilizes mathematical and statistical methods to process and analyze policy documents, with the aim of extracting valuable insights from large volumes of policy texts to provide a scientific basis for policy formulation, evaluation, and optimization . Yang et al. (35) applied this method to analyze “Internet + Healthcare” policies, identifying four key focus areas: facilities, technology, services, and management. The study also identified implementation challenges and recommended that future policies expand participation, strengthen IT support, and clarify responsibility allocation to fully leverage the potential of “Internet + Healthcare” in enhancing service efficiency and equity.

As health and medical management policies continue to evolve, policy text analysis has become a prominent research avenue (36, 37). The PMC index model, an effective text analysis tool, has been widely applied in public policy and health policy research (38, 39). It assesses the efficacy of policy formulation and its potential repercussions during implementation by analyzing the content, structure, and language of policy texts. For example, Duan et al. (40) utilized the PMC index model, adopting a policy diffusion perspective, to study China's Long-Term Care Insurance (LTCI) policy. They constructed a novel policy diffusion analysis framework, extending LTCI policy research from qualitative to quantitative domains. The study focused on analyzing the impact of policy quality and consistency on diffusion characteristics and their underlying causes. Zhang et al. (41) conducted an examination of 13 central government policies on traditional Chinese medicine (TCM) for public health emergencies issued in the past two decades, and generated specific recommendations: integrating traditional medicine into emergency systems requires strengthening legal frameworks, enhancing multidisciplinary team development, and prioritizing the generation of clinical evidence for integrated Chinese and Western medicine. These suggestions offer a definitive guide for TCM's further development within public health emergency response systems.

In chronic disease management policy research, the PMC model is applicable for analyzing prevention and control objectives, implementation measures, and effectiveness evaluations proposed in various policy documents (42, 43). However, current domestic research on PMC text analysis of smart chronic disease management policies is still comparatively limited. PMC text analysis facilitates a holistic assessment of policy texts across multiple dimensions, such as completeness, consistency, and feasibility, thereby providing more thorough and in-depth grounds for policy optimization. Therefore, conducting PMC text analysis of China's smart chronic disease management policies carries substantial theoretical and practical significance.

## 3 Methods

### 3.1 Policy text mining

**Figure 1:**
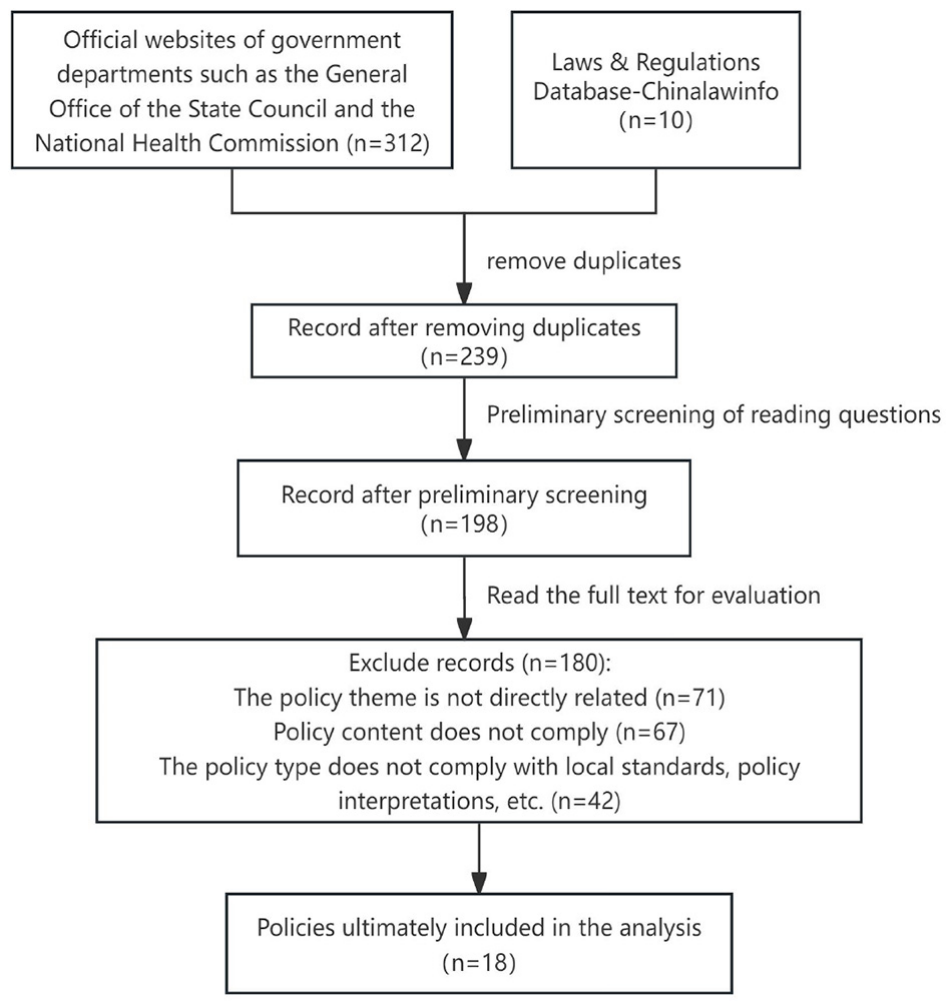
Policy retrieval process diagram.

In 2015, the State Council issued the Guiding Opinions on Actively Promoting the “Internet + Initiative,” explicitly proposing to leverage the internet's role as an innovation driver and promote its deep integration with all economic and social sectors, including healthcare. This laid the policy foundation for applying internet technology to chronic disease prevention and control. In 2017, the State Council further formulated the “China Chronic Disease Prevention and Control Medium- and Long-Term Plan (2017–2025),” emphasizing the importance of chronic disease prevention and control. Guided by the two core policies, this study systematically identifies national policy documents published from 2015 to 2025 by searching the Peking University Law Database and Chinese government websites using the core query: (“chronic disease” OR “chronic diseases”) AND (“Internet +” OR “smart healthcare” OR “digital health”). The search results were screened in strict accordance with the following criteria: clearly excluding provincial and below local policies, and only including national level planning, opinions and other normative documents issued by the State Council and its ministries and commissions, which are highly relevant to the theme, to ensure that the research focuses on the national top-level design and ensure the comparability of the samples, policy retrieval process diagram (Figure 1).

To ensure the objectivity of the scoring, two researchers independently evaluated 18 policies using PMC indicators, and calculated the Cohen's kappa coefficient to be 0.727. According to Landis and Koch's criteria (44), this indicates a high degree of consistency among raters.

For policy text analysis, this study employed ROST CM 6 and Gephi. The process began with the ROST CM 6 tool processing the 18 policy texts to perform word frequency statistics. Subsequently, a co-occurrence matrix was constructed based on the top 100 high-frequency words, applying a co-occurrence threshold of ≥3 to filter out generic administrative terms and retain associations between core concepts. This matrix was then imported into Gephi for semantic network analysis and thematic clustering. In Gephi, an edge weight filter of ≥5 was applied. For network visualization, the Fruchterman Reingold layout algorithm (with parameters set to Speed = 1 and Gravity = 12) was employed, with its parameters optimized to produce a clear representation.

### 3.2 PMC index model

The Polynomial Modeling and Comparison (PMC) Index model, originally developed by Estrada, focuses on quantifying policy internal consistency and implementation effectiveness through a systematic evaluation framework. This model first decomposes policy objectives into quantifiable key variables and establishes corresponding evaluation indicators for each variable, thereby forming a comprehensive policy assessment system. This study employs the PMC Index model to conduct a quantitative evaluation of chronic disease management policy texts. The PMC index model construction process comprises four main steps:

(1) Variable Classification and Parameter Identification: first, determine core evaluation dimensions and subdivide specific indicators, clarifying the weighting and scoring criteria for each parameter. According to the policy evaluation framework proposed by scholars such as Estrada (45), existing research on the PMC index model (46–49), and the results derived from high-frequency word analysis and semantic clustering topic analysis, the primary and secondary indicators for the chronic disease management policy evaluation system were constructed.

Assign binary values to secondary variables using (Equations 1, 2).


$$\begin{array}{lll} \text{X} & \sim & \text{N}\left[0,1\right] \end{array}$$ (1)

$$\begin{array}{lll} \text{X} & = & \left\{\text{XR}:\left[0\sim 1\right]\right\} \end{array}$$ (2)

(2) Establishing a Multi-Input Output Table: structuring parameter data to form a correspondence table between inputs and outputs ensures systematic evaluation.
(3) PMC Index Calculation: compute the PMC index using the “policy modeling” package in R. Integrate weighted scores of each indicator via a polynomial function to generate a PMC index ranging from 0 to 10, where higher values indicate superior overall performance. Following the PMC index calculation methodology, the values for corresponding primary variables were computed using Equation 3 based on variable parameters in the multi-input-output table. The PMC index for each policy was then calculated via Equation 4. In Equation 3, *t* denotes primary variables and *j* denotes secondary variables. In Equation 4, *r* represents the policy identifier and ~ denotes secondary variables.

$$\begin{array}{l} X_{t}\left(\frac{\sum_{j=1}^{n}X_{t,j}}{n}\right)t=1,2,3,4,5,6,7,8,9,10 \end{array}$$ (3)

$$\begin{array}{l} PMCr=\\ \left[\begin{array}{c} X_{1}\left(\sum_{i=1}^{2}\frac{X_{1i}}{2}\right)+X_{2}\left(\sum_{j=1}^{3}\frac{X_{2j}}{3}\right)+X_{3}\left(\sum_{k=1}^{4}\frac{X_{3k}}{4}\right)+\\ X_{4}\left(\sum_{l=1}^{5}\frac{X_{4l}}{5}\right)+X_{5}\left(\sum_{m=1}^{4}\frac{X_{5m}}{4}\right)+X_{6}\left(\sum_{n=1}^{8}\frac{X_{6n}}{8}\right)+\\ X_{7}\left(\sum_{o=1}^{8}\frac{X_{7o}}{8}\right)+X_{8}\left(\sum_{p=1}^{5}\frac{X_{8p}}{5}\right)+X_{9}\left(\sum_{q=1}^{4}\frac{X_{9q}}{4}\right)+X_{10} \end{array}\right] \end{array}$$ (4)

Then, this study referenced existing policy evaluation grading standards (42) to classify policy quality into five tiers: unacceptable, acceptable, good, excellent, and perfect. The criteria for tier classification are as follows in Table 1: when the PMC index falls between 9 and 10, policy consistency is highest, classified as “perfect”; scores between 8 and 8.99 are rated “excellent”; scores between 6 and 7.99 are classified as “good”; scores between 4 and 5.99 indicate weaker policy coherence and are rated “acceptable”; and scores between 0 and 3.99 denote very weak policy coherence, rated “poor.”

(4) PMC Surface Map Construction: visualize the calculated PMC indices using a three-dimensional surface. First, construct a 3 × 3 matrix based on the PMC indices of the top nine key variables for each policy. The PMC matrix expression (Equation 5) is as follows: This matrix excludes X10, primarily because the X10 values for all 18 policies are 1, rendering them irrelevant for surface mapping. Additionally, this exclusion maintains the symmetry of the PMC surface. Subsequently, specialized plotting software generates the PMC surface plot.

$$\begin{array}{c} PMCr=\left[\begin{array}{c} X_{1}\;X_{2}\;X_{3}\\ X_{4}\;X_{5}\;X_{6}\\ X_{7}\;X_{8}\;X_{9} \end{array}\right] \end{array}$$

**Table 1:** Policy scoring levels.

**Evaluation criteria**	**Poor**	**Acceptable**	**Good**	**Excellent**	**Perfect**
PMC score	0–3.99	4–5.99	6–7.99	8–8.99	9–10

## 4 Results

### 4.1 Results of policy text mining

Through systematic screening, 18 policy documents related to smart healthcare for chronic disease prevention and control were collected and subjected to quantitative analysis and evaluation using the PMC index model. Detailed information including policy release years, issuing authorities, issue numbers, and policy titles is presented in Table 2.

**Table 2:** Summary of samples for evaluating the effectiveness of chronic disease policies.

**No**.	**Name**	**Issuing authority**	**Issue number**	**Year**
P1	Interim Measures for the Management of Generative Artificial Intelligence Services	National Cyberspace Administration of China, National Development and Reform Commission, Ministry of Education, Ministry of Science and Technology, Ministry of Industry and Information Technology, Ministry of Public Security, State Administration of Radio, Film and Television	No. 15	2023
P2	Notice on Issuing the 14th Five Year Plan for National Health Informatization	National Health Commission, National Administration of Traditional Chinese Medicine, National Bureau of Disease Control and Prevention	National Health Planning [2022] No. 30	2023
P3	Opinions on Further Improving the Medical and Health Service System	General Office of the Communist Party of China Central Committee and General Office of the State Council	Zhongbanfa [2023] No. 10	2023
P4	Notice of the General Office of the State Council on Issuing the 14th Five Year Plan for National Health	General Office of the State Council	Guobanfa [2022] No. 11	2022
P5	Notice on Printing and Distributing the Detailed Rules for the Supervision of Internet Diagnosis and Treatment (for Trial Implementation)	Office of the National Health Commission and Office of the State Administration of Traditional Chinese Medicine	National Health Office Medical Development [2022] No. 2	2022
P6	Notice of the State Council on Issuing the 14th Five Year Plan for the Development of the Digital Economy	The State Council	Guofa [2021] No. 29	2022
P7	Notice from the Ministry of Industry and Information Technology, the Ministry of Civil Affairs, and the National Health Commission on Issuing the Action Plan for the Development of Smart Health and Older Adult Care Industry (2021–2025)	Ministry of Industry and Information Technology, Ministry of Civil Affairs, Health Commission	Ministry of Industry and Information Technology Lian Electronics [2021] No. 154	2021
P8	Notice of the General Office of the State Council on Issuing the “14th Five Year Plan” for National Medical Security	General Office of the State Council	Guobanfa [2021] No. 36	2021
P9	Opinions of the National Healthcare Security Administration on Optimizing Convenient Services in the Medical Insurance Field	Healthcare Security Administration	Medical Insurance Issue [2021] No. 39	2021
P10	Notice on Deepening the “Five Ones” Service Action of “Internet + Medical Health”	National Health Commission, National Medical Security Administration, and National Administration of Traditional Chinese Medicine	National Health Planning [2020] No. 22	2020
P11	Notice from the General Office of the Ministry of Industry and Information Technology and the General Office of the National Health Commission on Further Strengthening the Capacity Building of Remote Medical Networks	Office of the Ministry of Industry and Information Technology, Office of the Health Commission	MIIT Unicom Letter [2020] No. 251	2020
P12	Guiding Opinions of the National Medical Security Bureau on Improving the “Internet +” Medical Service Price and Medical Insurance Payment Policy	National Healthcare Security Administration	Medical Insurance Issue [2019] No. 47	2019
P13	Notice of the Bureau of Traditional Chinese Medicine of the Health Commission on Printing and Distributing Three Documents Including the Administrative Measures for Internet Diagnosis and Treatment (for Trial Implementation)	Traditional Chinese Medicine Bureau of the Health Commission	Guowei Medical Development [2018] No. 25	2018
P14	Opinions on Promoting the Development of “Internet ++Medical Health”	General Office of the State Council	Guobanfa [2018] No. 26	2018
P15	China's Medium - and Long Term Plan for the Prevention and Control of Chronic Diseases (2017–2025)	General Office of the State Council	Guobanfa [2017] No. 12	2017
P16	Notice of the General Office of the National Health and Family Planning Commission on Issuing the Management Measures for the Construction of National Chronic Disease Comprehensive Prevention and Control Demonstration Zones	Office of the National Health and Family Planning Commission	National Health Commission [2016] No. 44	2016
P17	Guiding Opinions on Promoting and Standardizing the Development of Health and Medical Big Data Applications	General Office of the State Council	Guobanfa [2016] No. 47	2016
P18	Guiding Opinions of the State Council on Actively Promoting the Action of “Internet +”	The State Council	Guofa [2015] No. 40	2015

**Figure 2:**
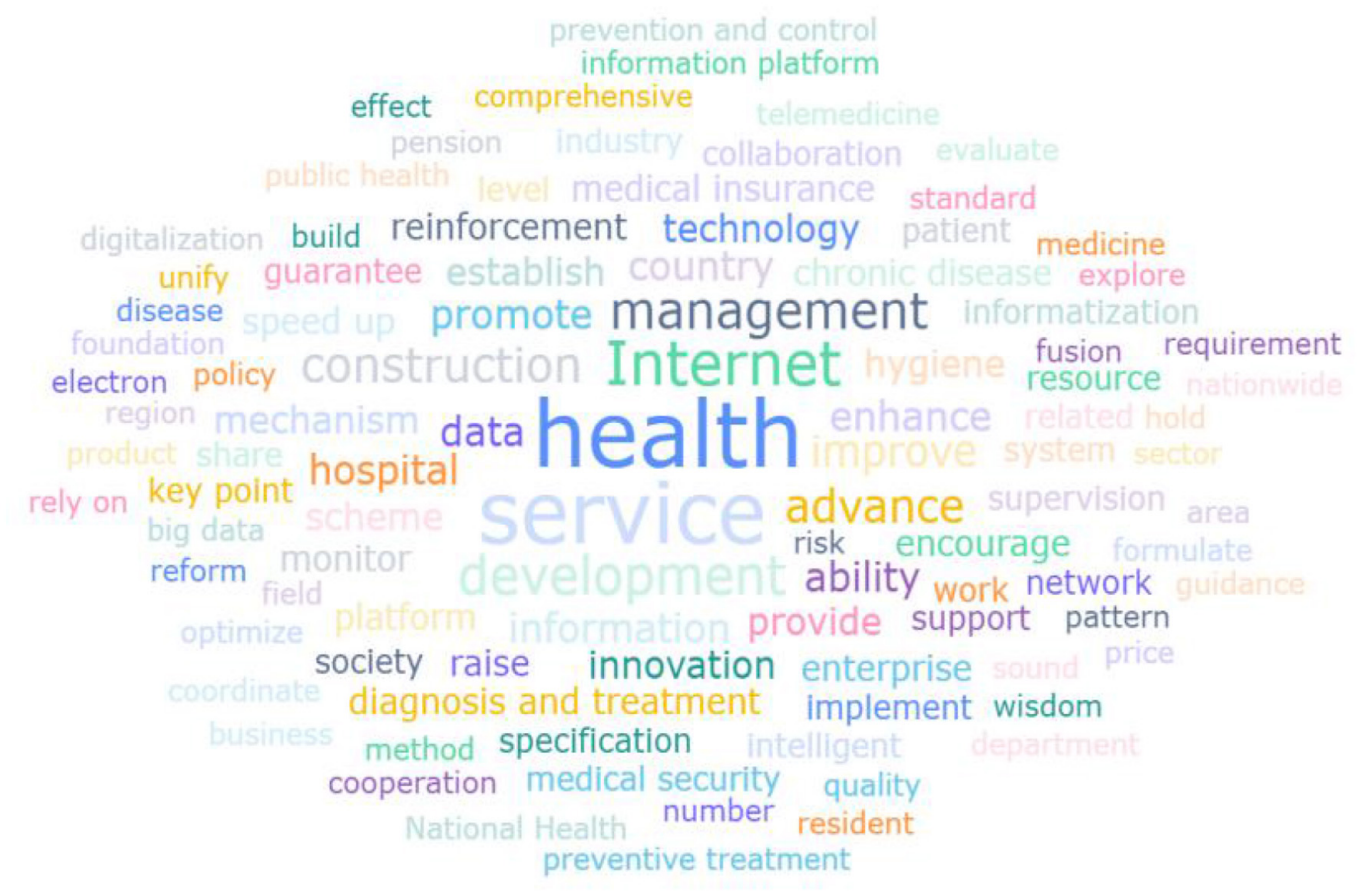
Frequency words in chronic disease policy.

The word frequency analysis reflects keywords frequently mentioned by policymakers. High-frequency terms extracted from policy texts facilitate deeper understanding of the priorities and objectives outlined in chronic disease management policies. By removing irrelevant and generic terms from this study, high-frequency words were extracted for word frequency analysis (Table 3), and visually analyze the relevant high-frequency words (Figure 2).

**Table 3:** Top 100 high-frequency word list.

**Words**	**Frequency**	**Words**	**Frequency**	**Words**	**Frequency**	**Words**	**Frequency**
Health	1,173	Monitor	191	Medical security	138	Hold	107
Service	1,119	Diagnosis and treatment	190	Industry	135	Wisdom	107
Internet	674	Platform	189	Fusion	132	National health	106
Development	518	Raise	189	Field	131	Explore	104
Management	449	Medical insurance	188	Policy	130	Department	103
Construction	423	Enterprise	187	Public health	128	Disease	101
Advance	370	Speed up	184	Build	127	Formulate	101
Improve	334	Support	171	Big data	125	Information platform	101
Data	296	Supervision	169	Standard	123	Evaluate	101
Enhance	293	Work	165	Sound	123	Risk	99
Promote	289	System	165	Reform	122	Digitalization	97
Information	287	Specification	162	Optimize	120	Unify	95
Ability	280	Informatization	159	Method	120	Product	94
Country	272	Guarantee	158	Telemedicine	119	Electron	93
Hospital	272	Related	158	Pattern	116	Price	92
Hygiene	258	Implement	156	Pension	115	Preventive treatment	92
Innovation	255	Intelligent	156	Comprehensive	115	Business	91
Establish	245	Resource	155	Sector	113	Requirement	90
Provide	240	Share	153	Number	113	Area	90
Scheme	236	Society	152	Foundation	112	Resident	90
Technology	228	Patient	149	Quality	111	Guidance	89
Mechanism	228	Key point	147	Coordinate	111	Effect	88
Encourage	217	Level	145	Cooperation	110	Prevention and control	88
Chronic disease	205	Network	143	Region	110	Rely on	87

**Figure 3:**
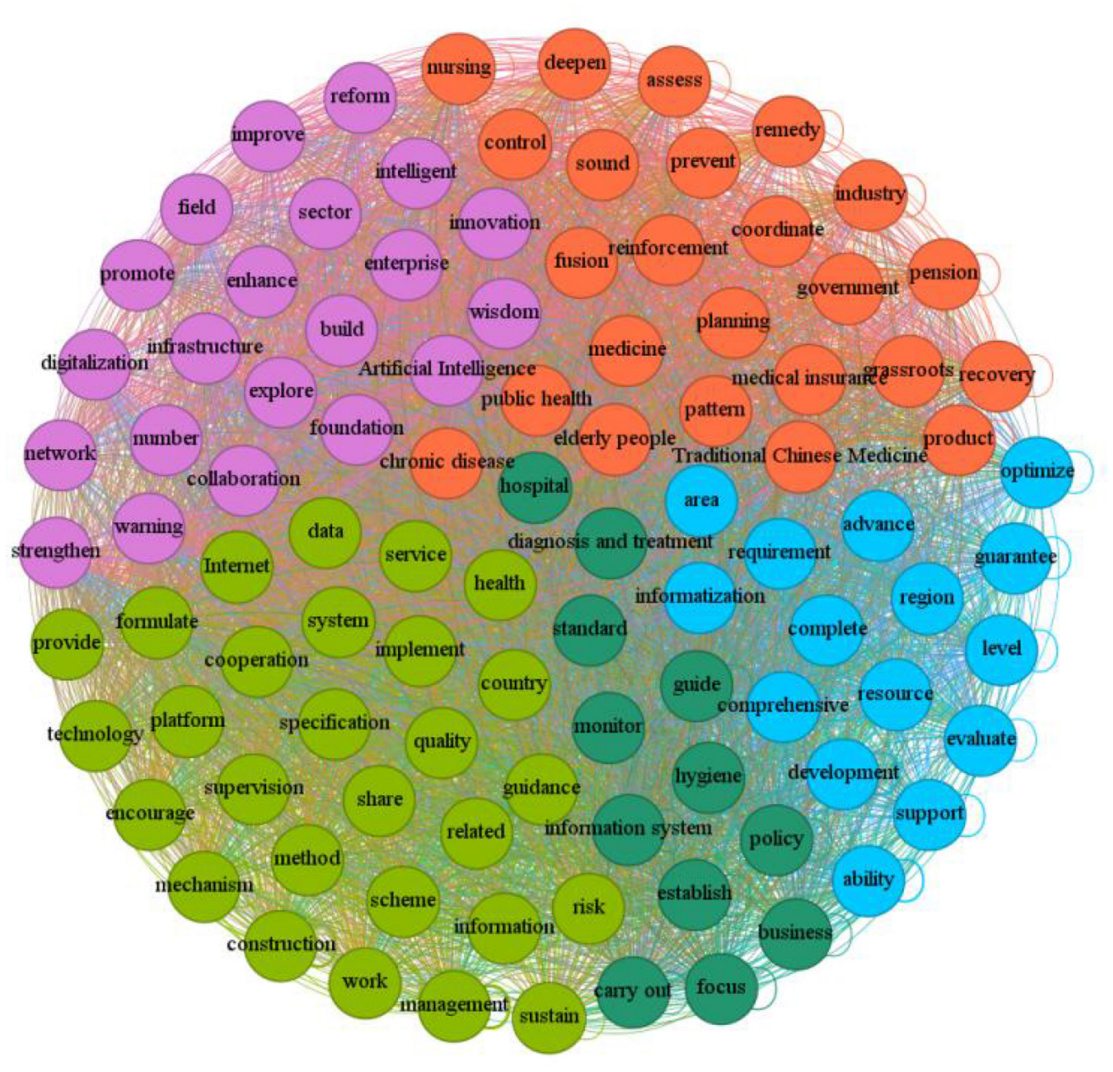
Thematic clustering map of semantic network.

The co-occurrence network clustering diagram of high-frequency words serves as a visual tool in text analysis, intuitively presenting lexical associations and thematic structures. It represents high-frequency words in the text as “nodes,” uses “lines” to indicate co-occurrence relationships between words, and forms “clusters” from closely connected nodes, which correspond to the core themes of the text. Words within the same cluster are highly semantically related due to frequent co-occurrence, collectively pointing to a specific theme. Overall, this helps researchers quickly identify core discussion directions and intrinsic logical connections from massive amounts of text. From the extracted high-frequency words, we obtained a co-occurrence matrix vocabulary. Based on this, we generated a network co-occurrence diagram for high-frequency words in Figure 3.

### 4.2 Results of indicator system for policies

This framework comprises 10 primary variables and 48 secondary variables. Primary variables include: Type of policy (X1), Policy timeliness (X2), Policy areas (X3), Policy tool (X4), Policy focus (X5), Policy level (X6), Policy theme (X7), Policy guarantee (X8), Policy evaluation (X9), and Policy disclosure (X10). Each primary variable is subdivided into several secondary variables to ensure comprehensive inclusion of relevant variables without omitting potential independent variables. All secondary variables are equally weighted and assigned binary values (involved = 1, not involved = 0). The complete evaluation indicator system is presented in Table 4.

**Table 4:** Scoring criteria for secondary variables.

**Primary indicators**	**Secondary indicators**	**Variables**	**Scoring criteria**	**Reference source**
Type of policy	X1	(X1:1) Prediction	1. Is the policy predictive?	Guo et al. (54)
		(X1:2) Regulation	2. Does the policy involve regulatory content?	Estrada (45)
		(X1:3) Suggestion	3. Does the policy provide recommendations?	
		(X1:4) Description	4. Does the policy have descriptive content?	
		(X1:5) Guidance	5. Does the policy provide guidance?	
Policy timeliness	X2	(X2:1) Short term acute response	1. Does the policy involve short-term emergency content (3 years)	Liu et al. (55)
		(X2:2) Mid term	2. Does the policy involve mid-term content (3–5 years)	
		(X2:3) Long term development potential	3. Does the policy involve long-term development planning (≥ 5 years)	
Policy areas	X3	(X3:1) Medical service field	1. Does the policy involve medical services?	Mayes et al. (56)
		(X3:2) Health management field	2. Does the policy involve health management content?	Yang (57)
		(X3:3) Information technology field	3. Does the policy involve information technology content?	
		(X3:4) Social security field	4. Does the policy involve social security content?	
Policy tool	X4	(X4:1) Construction type	1. Does the policy have a constructive effect?	Zhang et al. (41)
		(X4:2) Guidance type	2. Does the policy have a guiding role?	
		(X4:3) Standard type	3. Does the policy have a regulatory effect?	
		(X4:4) Service oriented type	4. Does the policy have a service-oriented approach?	
		(X4:5) Market oriented type	5. Does the policy have a market-oriented approach?	
Policy focus	X5	(X5:1) Chronic Disease Health Management	1. Does the policy focus on chronic disease health management?	High-frequency words
		(X5:2) Application of the Internet and informatization in chronic disease management	2. Does the policy emphasize the application of the Internet and informatization in chronic disease management?	
		(X5:3) Medical security and medical insurance support	3. Does the policy emphasize medical security and medical insurance support?	
		(X5:4) Multi party collaboration and cooperation	4. Does the policy emphasize multi-party collaboration and cooperation?	
		(X5:5) Technological Innovation and Smart Healthcare	5. Does the policy emphasize technological innovation and smart healthcare?	
		(X5:6) Public Health and Disease Prevention and Control	6. Does the policy emphasize public health and disease prevention and control?	
		(X5:7) Improvement of medical service capabilities	7. Does the policy emphasize the improvement of medical service capabilities?	
		(X5:8) Data sharing and standardization	8. Does the policy emphasize data sharing and standardization?	
		(X5:9) Industrial support and enterprise development	9. Does the policy emphasize industrial support and enterprise development?	
		(X5:10) Regulatory and evaluation mechanism	10. Does the policy emphasize regulatory and evaluation mechanisms?	
Policy level	X6	(X6:1) State Council	1. Does the policy recipient include the State Council?	Wei et al. (46)
		(X6:2) Various departments of the State Council	2. Does the policy recipient include all departments of the State Council?	
		(X6:3) Subordinate agencies of the State Council	3. Does the policy recipient include subordinate agencies of the State Council?	
		(X6:4) National Bureau managed by departments	4. Does the policy receptor include national bureaus managed by departments?	
Policy theme	X7	(X7:1) Technology driven	1. Does the policy theme include intelligent technology empowerment and industrial innovation?	Topic clustering
		(X7:2) Service integration	2. Does the policy focus on the prevention and control of chronic diseases and the construction of a health service system?	
		(X7:3) System mechanism	3. Does the policy theme involve the construction of information services and system mechanisms?	
		(X7:4) Resource Capability	4. Does the policy theme emphasize resource optimization and service capability enhancement?	
		(X7:5) Execution landing	5. Does the policy theme focus on the implementation and implementation of policies?	
		(X7:6) Other	6. Does the policy theme focus on other aspects of the policy?	
Policy guarantee	X8	(X8:1) Financial Security	1. Does the policy have financial security?	Wang et al. (47)
		(X8:2) Institutional safeguards	2. Does the policy have institutional safeguards?	
		(X8:3) Resource guarantee	3. Does the policy have resource guarantee?	
		(X8:4) Talent guarantee	4. Does the policy provide talent protection?	
		(X8:5) Incentive mechanism	5. Does the policy have incentive mechanisms?	
Policy evaluation	X9	(X9:1) Sufficient basis	1. Is the policy basis sufficient?	Gong et al. (48)
		(X9:2) Goal oriented	2. Is the policy objective guided?	
		(X9:3) Scientific plan	3. Is the policy proposal scientific?	
		(X9:4) Detailed planning	4. Is the policy planning detailed?	
		(X9:5) Clarify rights and responsibilities	5. Are policy responsibilities clear?	
Policy disclosure	X10	Policy Disclosure	1. Is the policy open to the public?	Cai et al. (58)

### 4.3 Results of PMC index model

#### 4.3.1 Temporal trends

We conducted Pearson correlation analysis on the policy year and its corresponding PMC index, and the results showed that there was no significant correlation between the policy year and the annual average PMC index (*r* = −0.349, *p* = 0.358). This indicates that China's intelligent chronic disease policy has remained relatively stable in the overall design framework and has not shown a clear trend of evolution over time.

#### 4.3.2 PMC index score

Combining the above results, the 10 primary variables and 48 secondary variables are integrated to construct a multi-input output table for the policy text. Specific results are presented in Table 5. That all evaluated policies meet the basic qualification standards. Among them, 72.2% demonstrate excellent performance, achieving a good rating; notably, 27.5% of policies are rated as outstanding due to their exceptional overall performance. The PMC indices of the 18 policies ranged from 6 to 8.5, with an average score of 7.31, indicating that the overall policy text ratings were “good” and “excellent.” The policy ranking is as follows: P14 > P2 > P17 > P4 > P15 > P7 > P3 > P10 > P8 > P18 > P6 > P16 > P13 > P11 > P5 > P12 > P1 > P9. Table 6 shows the comparison of PMC quantitative evaluation scores of different policy types, policies scored higher on average for X1 (Type of policy), X3 (Policy areas), X4 (Policy tool), X6 (Policy level), X7 (Policy theme), X8 (Policy guarantee), and X9 (Policy evaluation), while scoring lower on average for X2 (Policy timeliness) and X5 (Policy focus).

**Table 5:** Scores of 18 policies.

**Policy dimension**	**P1**	**P2**	**P3**	**P4**	**P5**	**P6**	**P7**	**P8**	**P9**	**P10**	**P11**	**P12**	**P13**	**P14**	**P15**	**P16**	**P17**	**P18**
X1 (Type of policy)	0.60	1.00	0.80	1.00	0.60	1.00	1.00	1.00	0.80	0.80	0.60	0.80	0.60	0.80	1.00	0.60	0.80	0.80
X2 (Policy timeliness)	0.33	0.67	1.00	0.67	0.33	0.67	0.33	0.67	0.67	0.67	0.33	0.67	0.67	1.00	0.67	0.67	0.67	0.33
X3 (Policy areas)	0.25	1.00	1.00	1.00	0.75	0.25	1.00	1.00	0.75	1.00	0.50	0.75	0.50	1.00	1.00	1.00	1.00	1.00
X4 (Policy tool)	1.00	1.00	0.80	1.00	1.00	1.00	1.00	1.00	0.80	1.00	0.80	1.00	1.00	1.00	1.00	0.80	1.00	1.00
X5 (Policy focus)	0.60	0.80	0.50	0.60	0.60	0.50	0.90	0.30	0.30	0.60	0.50	0.50	0.60	0.70	0.60	0.50	0.90	0.50
X6 (Policy level)	1.00	1.00	1.00	1.00	0.75	1.00	0.75	1.00	0.25	0.75	0.75	0.25	0.75	1.00	1.00	0.50	1.00	1.00
X7 (Policy theme)	0.67	1.00	0.83	0.83	1.00	0.83	1.00	0.67	0.83	1.00	1.00	1.00	1.00	1.00	0.83	0.83	1.00	1.00
X8 (Policy guarantee)	0.60	1.00	1.00	1.00	0.20	1.00	1.00	1.00	0.60	1.00	0.80	0.20	0.20	1.00	1.00	1.00	1.00	1.00
X9 (Policy evaluation)	1.00	1.00	1.00	1.00	1.00	1.00	1.00	1.00	1.00	1.00	1.00	1.00	1.00	1.00	1.00	1.00	1.00	1.00
PMC index	**6.05**	**8.47**	**7.93**	**8.10**	**6.23**	**7.25**	**7.98**	**7.63**	**6.00**	**7.82**	**6.28**	**6.17**	**6.32**	**8.50**	**8.10**	**6.90**	**8.37**	**7.63**
Policy effectiveness level	**G**	**E**	**G**	**E**	**G**	**G**	**G**	**G**	**G**	**G**	**G**	**G**	**G**	**E**	**E**	**G**	**E**	**G**

G, good; E, excellent.

**Table 6:** Comparison of PMC scores of different policy types.

**Policy type**	**PMC scores**
X1 (Type of policy)	0.81
X2 (Policy timeliness)	0.61
X3 (Policy areas)	0.82
X4 (Policy tool)	0.96
X5 (Policy focus)	0.58
X6 (Policy level)	0.82
X7 (Policy theme)	0.91
X8 (Policy guarantee)	0.81
X9 (Policy evaluation)	1.00

This study categorizes X1, X4, and X6 as structural dimensions and X2 and X5 as content dimensions, and calculates the mean values of the two dimensions for 18 policy samples. From a single sample perspective, the structural dimension mean (range 0.62–1.00) of all policies is higher than their content dimension mean (range 0.42–0.85), indicating that policies generally outperform content elements in the design of structural elements. Overall, the total mean of the structural dimension is 0.84, and the total mean of the content dimension is 0.61, further confirming that policy texts generally focus more on the construction of structural elements.

**Figure 4:**
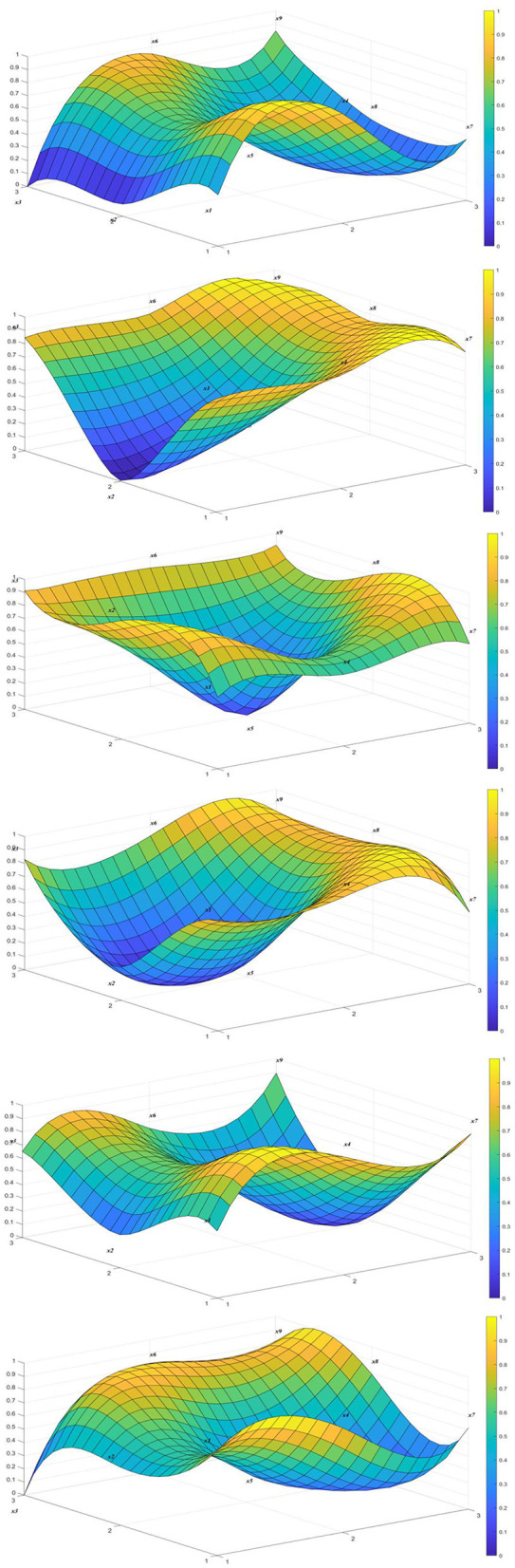
Chronic disease policy excellence effectiveness surface (P1–P6).

In order to present the relevant policy situation more intuitively, we selected six policies from the 18 policies for graphic display, as shown in Figure 4.

Moreover, the convexity/concavity and color variations within the PMC surface plot intuitively reflect performance disparities across policy indicator dimensions. Convex regions (warm color scheme) indicate superior policy performance and higher scores in that metric, signifying significant effectiveness of related measures. Concave areas (cool color scheme) reveal deficiencies in that dimension, necessitating targeted improvements. This visualization method clearly displays index changes under different parameter combinations, enabling policymakers to observe surface undulations and better identify policy strengths and weaknesses. Consequently, it facilitates a scientific assessment of policy performance across all dimensions.

**Figure 5:**
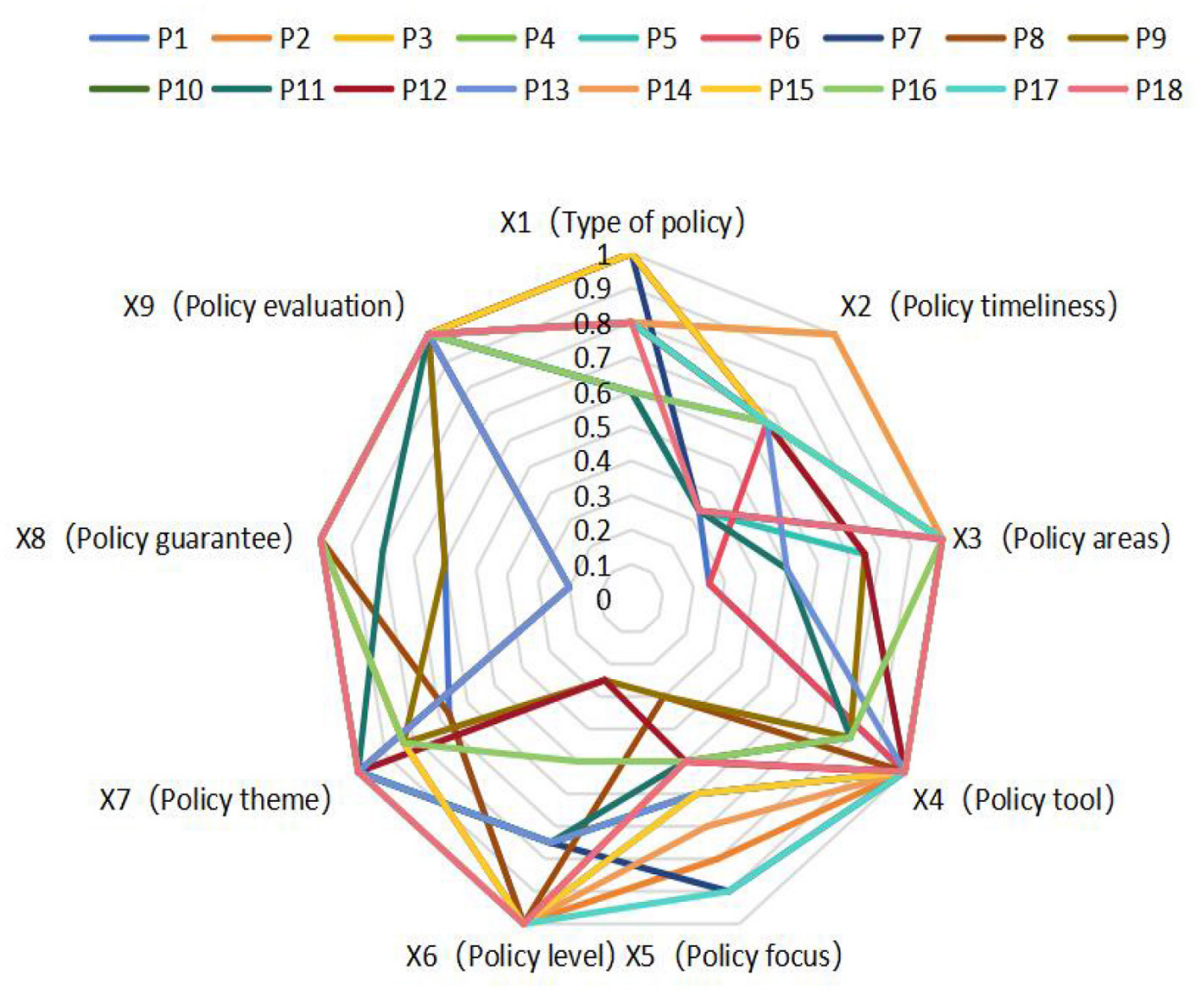
Radar chart of chronic disease policies.

The radar chart in Figure 5 visually presents the scores for each policy's primary variables, clearly highlighting their strengths and weaknesses.

## 5 Discussion

### 5.1 Discussion for policy text mining

The word frequency and thematic clustering analysis of 18 intelligent chronic disease management policy texts not only systematically reveals the current policy priority direction, but also highlights the potential tension between macro narrative and micro implementation of policies, providing empirical evidence for understanding the internal logic and optimization path of policy frameworks in this field. Word frequency analysis shows that “health,” “service,” “Internet,” “data,” “construction,” “management,” “promotion” and other words constitute the core of the policy text. Among them, the high-frequency co-occurrence of “health” and “service” clarifies the value orientation of policies with the fundamental goal of improving the health level of the entire population and optimizing service supply as the core path, which is consistent with the core concept of “putting people's health at the center” in the “Healthy China 2030” strategy. “Internet” and “data” have been repeatedly emphasized as key enabling tools, which confirms that digital technology is regarded as the core engine driving the innovation of chronic disease management model. At the same time, the prominent use of dynamic terms such as “development,” “promotion,” and “construction” collectively outlines a proactive policy framework that emphasizes technology driven, system building, and continuous improvement.

Further theme clustering extracts five core themes from the semantic content of the text, including “Empowering with Intelligent Technologies and Fostering Industrial Innovation,” “Chronic Disease Prevention and Control with Health Service System Development,” “Information Services and System Mechanism Development,” “Resource Optimization and Service Capacity Enhancement”, and “Policy Implementation and Execution.” These themes are interrelated, forming a complete policy narrative chain from “top-level strategic design (technology empowerment, system integration)” to “middle-level support construction (information mechanism, resource optimization)”, and then touching on “grassroots implementation.” This structure clearly indicates that the current policy design has distinct systematic and comprehensive characteristics, aiming to promote smart chronic disease management through multi-dimensional collaboration.

However, meticulous text analysis also exposes the potential disconnect between policy intentions and actual implementation environments, pointing to key optimization spaces: the frequent emergence of the term “construction” and the theme of “information services and institutional mechanism construction,” collectively indicating a high tilt of policy resources toward infrastructure such as information platforms and data systems. In contrast, statements related to service evaluation, quality supervision, and multi-party governance are relatively weak. This implies that there may be a risk of policies that prioritize upfront investment over sustainable operation and efficiency governance, which could lead to difficulties in converting construction achievements into sustainable service capabilities. Although verbs such as “promote” and “enhance” clearly indicate policy direction, there is insufficient detailed description of the specific “implementation process,” “division of responsibilities,” and “collaborative mechanism.” As an independent theme, the text intensity and specificity of “policy implementation and execution” are significantly inferior to other targeted themes. This reflects the gap between setting grand visions and providing clear and actionable implementation roadmaps in policies, which can lead to inconsistent understanding and difficulty in coordination among the executive level, resulting in implementation gaps. Although concepts such as “artificial intelligence” and “big data” are widely embedded in various topics, there is a lack of in-depth discussions specifically addressing governance issues such as “algorithm ethics,” “data security,” “privacy protection,” and “digital inclusion.” This indicates that in the process of promoting the integration and application of technology, the corresponding adaptive governance framework and ethical norms have not been deepened synchronously, which may become a potential bottleneck for future technology implementation and public trust.

Examining the above findings within the global digital health governance landscape reveals both consensus and distinctive features. China's policy framework emphasizes the use of data to enhance service accessibility and governance efficiency, which is highly consistent with the World Health Organization's (WHO) Global Digital Health Strategy (2020–2025) and the core principles of digital health governance advocated by the Organization for Economic Cooperation and Development (OECD) (49, 50). However, China's path shows a unique top-level design feature of “national leadership and systematic promotion.” It deeply integrates chronic disease management into the overall situation of medical reform through national strategies such as “Internet plus+health care,” and its policy is particularly holistic and coordinated. Compared with more focused strategies such as Germany (focusing on data privacy and security frameworks) or Tanzania (focusing on community empowerment and basic access) (51), China's current policies tend to systematically address broad challenges such as “inclusive services” and “basic empowerment.” This comprehensive path has advantages in rapid layout and the formation of economies of scale, but it also puts higher demands on the precision of policies, the meticulousness of implementation, and the depth of special governance.

Therefore, the policy layout revealed in this study not only confirms China's practical path of “technology as the support and service as the purpose,” but also points out the future optimization direction-while maintaining the advantages of top-level design, we need to focus on strengthening the implementation of monitoring and multiple co treatment mechanisms, so as to build a more flexible and inclusive chronic disease treatment system.

### 5.2 Discussion for comprehensive performance analysis of policy texts

Through Pearson correlation analysis of policy years and PMC index, the results showed no significant correlation between the two. This indicates that the average PMC index of policies does not show a significant linear time trend, and the index fluctuates greatly in each year, reflecting that under this evaluation system, the consistency of policy quality in different years still needs to be strengthened, and there is still room for improvement in the scientific and normative design of policies. This phenomenon may be due to the different dimensions of policy emphasis in different years (such as technology application, service processes, guarantee mechanisms, etc.), resulting in fluctuations in PMC scores without forming a sustained upward trend.

The comprehensive performance analysis of the policy text shows that there are obvious systematic differences in the scores of each dimension, which deeply reflects the internal logic and focus of the current intelligent chronic disease management policy system. The dimensions of policy tool (X4 = 0.96), policy theme (X7 = 0.91), policy guarantee (X8 = 0.81), and policy evaluation (X9 = 1.00) generally received high scores, jointly outlining a standardized, comprehensive, and multi tool macro policy framework, reflecting the strong systematic construction ability at the top-level design level. In contrast, the scores of the two dimensions of policy focus (X5 = 0.58) and policy timeliness (X2 = 0.61) are significantly lower, constituting a shortcoming in the overall evaluation. The former indicates that while the policy text covers a wide range of fields, the prioritization of core challenges and the in-depth explanation of key measures are insufficient; The latter reveals that the connection and dynamic adjustment mechanism between short-term actions, medium-term planning, and long-term strategies is still unclear.

The clustering distribution of high and low score dimensions is not accidental. It points to a noteworthy tendency in the policy-making process: investing more in pursuing completeness of form and breadth of content, while relatively weak in focusing on strategic depth and dynamic coordination of planning. High scoring dimensions tend to focus on describing what elements policies should include and how to provide support, reflecting strong supply side planning thinking; the low score dimension is precisely related to how policies determine key breakthroughs in complex and changing environments, and maintain resilience and adaptability over time. These are the key process factors that affect the ultimate effectiveness of implementation.

Therefore, the overall pattern of performance scores reveals that the advantage of existing policy texts lies in constructing a standardized and widely covered macro framework, laying a solid foundation for the development of intelligent chronic disease management. However, in order to translate the policy blueprint into solid results, future optimization directions need to focus on addressing existing shortcomings, shifting from emphasizing the comprehensiveness of the framework to simultaneously enhancing strategic focus, coordination of action sequences, and flexibility for cross period adaptation. This requires policy formulation not only to focus on the completeness of static content, but also to embed dynamic process management thinking, thereby promoting the entire system to move from having a good foundation to achieving precise and efficient operation.

### 5.3 Discussion for quantitative evaluation analysis of policies at different levels

Among these policies, P14, P2, P17, P4, and P15 were rated “excellent,” reflecting common characteristics of high-quality policies. First, they are underpinned by top-level strategic support. P2 and P4 serve as national-level guiding documents, positioning “health big data” and “internet-based healthcare” as core pathways for chronic disease management, ensuring policy direction and stability. Second, they deeply focus on technology empowerment and integrated innovation. P14 actively promotes the deep integration of internet technology with healthcare services. P17 provides data support for intelligent chronic disease management. High-frequency terms like “data interoperability,” “platform development,” and “technical standards” appear significantly more frequently in these two policies, reflecting a strong emphasis on technological convergence. Third, emphasizing specialized precision measures, P15 stands as the sole specialized policy, ensuring coordinated and mutually reinforcing policy components.

The remaining 13 policies achieved a “good” rating, indicating that China's smart chronic disease management policy framework has taken shape but still holds room for improvement. First, while policies cover a broad scope, they lack sufficient specialization (e.g., P6, P18). Second, interdepartmental coordination mechanisms are nascent with limited innovation-driving capacity (e.g., P7). Third, foundational standards have taken shape but supporting infrastructure remains weak (e.g., P5). Compared to “excellent” policies, these “good” policies fall short in three main areas: insufficient specialized policy supply (only P16 is specialized among the 13); lack of specific application scenarios for technologies (e.g., P1); and absence of incentive and safeguard mechanisms (e.g., P9).

P14 received the highest score of 8.5 and was rated as an “excellent” policy. P9 received the lowest score of 6, yet was still classified as “Good.” The disparity likely stems from the following factors: In terms of policy positioning, P14 possesses a more macro strategic orientation and comprehensive policy framework. Regarding technological innovation, P14 demonstrates stronger leadership, encouraging new service models and the deep application of emerging technologies. In contrast, P9 primarily focuses on migrating traditional medical insurance services online. Regarding implementation safeguards, P14 establishes more comprehensive supporting mechanisms and demonstrates broader radiating effects across multiple sectors. The key reason P9 maintains a “good” rating lies in its resolution of the public pain point of healthcare service accessibility.

### 5.4 Policy implications

This study employs a combined approach of text mining and PMC index analysis. The PMC Index scores for the 18 policies ranged from 6 to 8.5, with an average score of 7.31, reflecting overall advantages in policy design and implementation.

However, compared with existing literature, this study reveals potential problems in the implementation of chronic disease management policies in China. Xue et al. (52) pointed out systemic deficiencies in China's chronic disease management policies, such as weak integration, unclear communication mechanisms in medical institutions, and lagging information platform construction. The analysis in this study shows that China's chronic disease management policies score higher in the dimensions of “policy tools” and “policy content,” but the score for “policy timeliness” is low. This highlights shortcomings in policy design and affects overall effectiveness, which is consistent with Xue et al.'s findings.

Li et al. (53) noted that the lack of long-term planning and flexibility in policy adjustments could affect sustainability and adaptability. This study echoes that point. In terms of “policy focus” and “policy timeliness,” the depth and flexibility of the policies are insufficient. Only 10 of the 18 policies have a long-term plan of 5 years or more, reflecting weaknesses in long-term sustainability and dynamic adjustment. Additionally, the lack of depth and focus in policy priorities poses further challenges.

Therefore, we propose the following suggestions:

Firstly, strengthen differentiated long-term planning and dynamic adjustment mechanisms. Based on the regional development level, formulate a long-term plan with hierarchical classification for 5 years or more, and clarify the phased goals: the developed eastern region focuses on the functional upgrading and deep application of smart chronic disease management platforms, the central region focuses on the construction of regional level chronic disease management centers and cross institutional data exchange, and the western region prioritizes the construction of smart infrastructure for grassroots medical institutions to fill gaps. At the same time, establish a normalized policy evaluation mechanism, rely on a stakeholder alliance composed of health administrative departments, medical institutions, research institutes, and patient groups, build regular feedback channels, and dynamically optimize planning content based on regional implementation effectiveness and people's livelihood needs.

Secondly, focus on key policy areas and clarify the precise implementation path of core technologies and services. Anchor the scenario application direction of core technologies such as AI, big data and IoT: support the research, development and promotion of AI algorithm in risk stratification and complication warning of chronic diseases such as hypertension and diabetes; Promote the popularization of IoT wearable devices in real-time monitoring of physical signs and medication reminders for chronic disease patients at home, especially for the older adults in rural areas, and develop low-cost and easy-to-use device adaptation solutions. Build a service system covering high-risk population screening, standardized diagnosis and treatment, and long-term health management around the entire process of chronic disease prevention, diagnosis, treatment, and rehabilitation, and connect it with medical insurance payment policies to include eligible smart chronic disease management services in the reimbursement scope.

Thirdly, establish a multi departmental collaborative governance mechanism to promote resource integration and benefit coordination. Establish a joint conference system led by the health department and involving multiple departments such as medical insurance, industry and information technology, finance, and civil affairs, clarifying the responsibilities of each department: the health department is responsible for policy standard formulation and clinical pathway standardization, the medical insurance department is responsible for payment method reform and cost control, the industry and information technology department is responsible for technical standards and industry support for smart medical equipment, and the finance department is responsible for special funding support for central and western regions and grassroots institutions. At the same time, we will establish a diversified collaboration model of “government guidance+market participation,” encourage private capital to participate in the construction and operation of smart chronic disease management platforms, promote medical institutions, research institutes, and technology enterprises to form industry university research innovation alliances, and accelerate the transformation of technological achievements.

Finally, improve the policy evaluation and supervision system that takes into account multiple dimensions. Construct a comprehensive evaluation framework that covers policy effectiveness, social benefits, economic efficiency, and implementation feasibility, incorporating core indicators such as regional adaptability, infrastructure coverage, patient satisfaction, and local fiscal affordability, fully considering political and economic factors such as economic development levels and medical resource endowments in different regions. Establish a dual supervision mechanism of “government supervision and third-party evaluation,” introduce independent third-party institutions to carry out policy implementation effect evaluation, and at the same time, open up public supervision channels to ensure transparency and fairness in the policy implementation process.

### 5.5 Limitations and future research

This study also has several limitations. Firstly, the PMC analysis method has technical limitations: the binary scoring method used for quantitative analysis is a necessary simplification of the complexity of policy provisions and may not reflect the differentiated importance of policy texts; Although the equal weight setting of analysis dimensions is based on the consideration of structural balance, its optimality needs further verification in subsequent research; In addition, the evaluation results lack external correlation testing with the actual implementation effect data of policies, mainly reflecting the characteristics of text content. Secondly, this study focuses on national level policies. Although it helps to grasp the macro strategic framework, it excludes provincial and local policies, which may introduce selection bias and make it difficult to fully reflect the huge differences in disease spectrum, resource endowment, and cultural customs in different regions of China. These factors profoundly affect the local adoption and implementation effectiveness of policies.

Future research can be improved in three areas: (1) Expand the scope to incorporate local policies into the analytical framework, examining the interplay between national and local policies in light of regional disease characteristics and economic-cultural variations to uncover local insights; (2) Adopt mixed-methods research combining qualitative approaches (e.g., surveys, in-depth interviews) with medical big data analysis to construct a “text-practice-outcome” three-dimensional evaluation model; (3) Establish dynamic tracking mechanisms using policy simulation techniques to predict policy evolution trends and develop intelligent monitoring systems for real-time implementation evaluation. It is recommended to prioritize multidisciplinary research integrating medicine, policy science, and data science methodologies to construct a Chinese-specific theoretical framework for intelligent chronic disease governance, thereby providing decision support for tiered diagnosis and treatment reforms.

## 6 Conclusions

This study conducted a systematic text evaluation and content analysis of China's national level smart chronic disease management policies by integrating text mining and PMC index models. The comprehensive evaluation shows that the current policy framework performs outstandingly in terms of tool diversity and thematic comprehensiveness, which is specifically reflected in policies such as “Notice on Issuing the 14th Five Year Plan for National Health Informatization,” which have detailed provisions for information technology tools, service system construction, and other aspects. The analysis also reveals systematic shortcomings in policy timeliness and policy focus. Multiple policy objectives are expressed in a grand and comprehensive manner, but there is a lack of clear guidance on the balance and ranking of parallel priorities such as “technological empowerment,” “medical insurance linkage,” and “grassroots capacity building,” which can easily lead to scattered implementation resources; Deployment related terms such as “construction” and “promote” frequently appear, while equivalent terms such as “evaluation” and “effect” have a relatively low proportion. At the textual level, there is a tendency to focus more on early-stage investment deployment and less on sustained operation and efficiency governance. This study proposes optimization suggestions from the source of policy text design to provide a specific path for bridging the gap in policy implementation. In response to the problem of unclear key areas, it is recommended that future policies clarify the time nodes and responsible parties of each task while retaining strategic comprehensiveness, and refine priority ranking; In response to the tendency of “heavy construction, light operation,” it is necessary to strengthen the institutionalized provisions of the operation mechanism of the intelligent chronic disease management platform, cross departmental data sharing rules, and cost-benefit evaluation cycle in the policy text, and incorporate operational efficiency into the core clauses of the policy.

At the methodological level, the contribution of this study lies in demonstrating the effectiveness of a hybrid research approach: by combining the quantitative PMC index model with qualitative text topic mining, it can not only standardize the structural completeness of policy design, but also deeply interpret the underlying priorities, potential tensions, and value orientations behind the text. A forward-looking and diagnostic evaluation framework applicable to the early stages of public policy has been developed, which can be effective in scenarios lacking large-scale implementation performance data. This framework provides a new tool for evaluating similar policy texts and lays a methodological foundation for exploring the correlation between policy text features and actual implementation effects in the future.

## Data Availability

The original contributions presented in the study are included in the article/supplementary material, further inquiries can be directed to the corresponding authors.
